# Molecular Structure–Affinity Relationship of Flavonoids in Lotus Leaf (*Nelumbo nucifera* Gaertn.) on Binding to Human Serum Albumin and Bovine Serum Albumin by Spectroscopic Method

**DOI:** 10.3390/molecules22071036

**Published:** 2017-06-23

**Authors:** Xiaosheng Tang, Ping Tang, Liangliang Liu

**Affiliations:** 1 Hubei Key Laboratory of Edible Wild Plants Conservation and Utilization & National Demonstration Center for Experimental Biology Education & College of Life Sciences, Hubei Key Laboratory of Pollutant Analysis & Reuse Technology, Hubei Normal University, Huangshi 435002, China; xiaoshengtang@yahoo.com; 2 Institute of Bast Fiber Crops, Chinese Academy of Agricultural Sciences, Changsha 410205, China; pingtang@yahoo.com; 3 School of Environmental Science and Engineering, Hubei Polytechnic University, Hubei Key Laboratory of Mine Environmental Pollution Control and Remediation, Huangshi 435003, China

**Keywords:** bovine serum albumin, flavonoids, fluorescence spectroscopy, human serum albumin, interaction, lotus leaf

## Abstract

Lotus leaf has gained growing popularity as an ingredient in herbal formulations due to its various activities. As main functional components of lotus leaf, the difference in structure of flavonoids affected their binding properties and activities. In this paper, the existence of 11 flavonoids in lotus leaf extract was confirmed by High Performance Liquid Chromatography (HPLC) analysis and 11 flavonoids showed various contents in lotus leaf. The interactions between lotus leaf extract and two kinds of serum albumins (human serum albumin (HSA) and bovine serum albumin (BSA)) were investigated by spectroscopic methods. Based on the fluorescence quenching, the interactions between these flavonoids and serum albumins were further checked in detail. The relationship between the molecular properties of flavonoids and their affinities for serum albumins were analyzed and compared. The hydroxylation on 3 and 3’ position increased the affinities for serum albumins. Moreover, both of the methylation on 3’ position of quercetin and the C_2_=C_3_ double bond of apigenin and quercetin decreased the affinities for HSA and BSA. The glycosylation lowered the affinities for HSA and BSA depending on the type of sugar moiety. It revealed that the hydrogen bond force played an important role in binding flavonoids to HSA and BSA.

## 1. Introduction

Flavonoids are a kind of antioxidant abundant in human diet and widespread in plants. Investigations of flavonoids from dietary have attracted great interests because of their bioactivities such as antioxidant activity, enzyme inhibitory activity, antitumor activity, and so on [1,2]. Most of their bioactivities are basically related to their antioxidant abilities. The structural differences of flavonoids significantly affect their absorption, metabolism, and activity in vivo [3,4]. While, the different structures of flavonoids come from various patterns of substitution through hydroxylation, methoxylation, sulfonation, acylation, prenylation, or glycosylation [5,6].

Lotus (*Nelumbo nucifera* Gaertn.) is a perennial aquatic plant widely distributed in eastern Asia, especially in China, India and Japan. The leaf, root, and seed of lotus were usually used as vegetables and medicines in China [7]. In recent years, the lotus leaf was becoming more and more popular as a kind of drink like tea in single form or as an ingredient in herbal formulations [8,9]. The lotus leaf was used in the treatment of sunstroke, diarrhea, and fever [10]. Modern studies also showed it possesses many pharmacological effects like anti-hyperlipidemia, anti-obesity, antioxidant, and anti-microbial activities [11,12,13]. Flavonoids are main functional components of lotus leaf and many of them were identified, including isoquercetin, hyperin, kaempferol, astragalin, myricetin, and so on [14,15,16,17].

Nowadays, investigations on the interaction between flavonoids and proteins garner great interest [18,19,20]. As one of the most abundant proteins in the circulatory system, serum albumins are the major transport protein in blood and can reversibly bind to small molecules like fatty acids, amino acids, drugs, and inorganic ions [21]. The bioavailability of many active compounds is related to the interaction and successive binding with serum albumins [22]. Forming stable binding complexes could be considered a suitable model for gaining various information of protein binding [23]. Human serum albumin (HSA) consists of a non-glycosylated single chain containing 585 amino acids with a molecular weight of 66,500 Da [24]. Three homologous domains (domains I–III), in turn divided into two subdomains (A and B), with multiple ligand-binding sites localized in each of these subdomains were described [20,25]. Many drugs bind to one of the two primary binding sites located in subdomains IIA and IIIA (Sudlow sites I and II, respectively) [26]. As the most prominent one, the IIA subdomain appears to be spacious. However, the IIIA site is smaller and less flexible and only structurally similar ligands could be accommodated. Bovine serum albumin (BSA) is a globular protein like HSA and has about 76% structural homology to HAS [27,28]. Considering the representative characteristics and the availability, the purified HSA and BSA are usually used as modeling proteins to investigate the interactions between small molecules and serum albumins. Binding of flavonoids to HSA was extensively studied using different fluorescence spectroscopy techniques [29,30,31,32,33]. The observed change in fluorescence enables the calculation of HSA-ligand complexes stability constants and also the distance between the ligand and Trp 214 [26]. Many studies reported the binding between flavonoids and serum albumins [34]. The structure–affinity relationship of flavonoids with serum albumins was studied as well. However, few reports have focused on the binding to the two kinds of serum albumins (HSA and BSA). The selection of flavonoids was various. Several reports were focused on the dietary flavonoids, while some reports selected different structures of flavonoids for comparison [35,36].

In this paper, 11 flavonoids were chosen as the investigation samples based on our previous research, which were reported existing in lotus leaf and showed similar structures. The contents of 11 flavonoids in lotus leaf extract were confirmed by High Performance Liquid Chromatography (HPLC) method. Furthermore, the interactions between lotus leaf extract and two kinds of serum albumins (human serum albumin (HSA) and bovine serum albumin (BSA)) were carried out by spectroscopic methods and then the interactions between these 11 flavonoids and serum albumins (HSA and BSA) were further investigated in detail. Then the relationship between the molecular properties of flavonoids and their affinities for HSA and BSA were analyzed and compared.

## 2. Results and Discussion

### 2.1. Analysis of Flavonoids in Lotus Leaf Extracts

**Figure 1:**
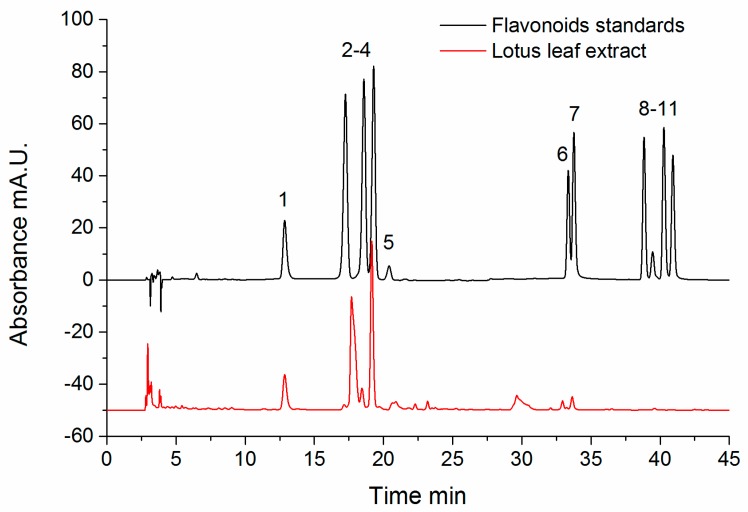
The chromatograms of standard mixture solution and lotus leaf extracts. The marked 11 flavonoids were astragalin (1), rutin (2), hyperoside (3), isoquercitrin (4), taxifolin (5), luteolin (6), quercetin (7), apigenin (8), naringenin (9), kaempferol (10), and isorhamnetin (11).

In order to understand the composition and content of selected flavonoids in lotus leaf extracts, the detail contents of 11 flavonoids in lotus leaf extracts were analyzed using HPLC. Eleven flavonoids—astragalin (1), rutin (2), hyperoside (3), isoquercitrin (4), taxifolin (5), luteolin (6), quercetin (7), apigenin (8), naringenin (9), kaempferol (10), and isorhamnetin (11)—were quantified with the standards in this study. The HPLC analysis provided a reproducible, well separated and detected method for the 11 flavonoid compounds [37]. The representative chromatograms of the standard mixture solution and lotus leaf extracts are illustrated in Figure 1.

It could be observed that 11 flavonoids were well separated at a retention time ranging from 12.86 min to 40.90 min. The quantitative analysis of each compound was accomplished by analyzing each standard compound with various concentrations. Based on the comparison of retention time, the contents of 11 flavonoids in lotus leaf extract were presented in Table 1. According to the HPLC results, astragalin, taxifolin, and isoquercitrin were abundant in lotus leaf extract (the contents of astragalin, taxifolin, and isoquercitrin were 13.347 ± 0.150, 10.597 ± 0.240, and 29.778 ± 0.180 mg/g of lotus leaf sample, respectively). However, apigenin, luteolin, kaempferol and isorhamnetin showed relatively low content in lotus leaf extract (less than 1.0 mg/g). Considering the selected flavonoids were reported by different literatures, the extraction using different solvents or different varieties and parts of lotus could result in the difference of contents of these flavonoids [38,39,40,41].

### 2.2. Quenching Effects of Lotus Leaf Extract and Flavonoids on HSA and BSA Fluorescence

**Figure 2:**
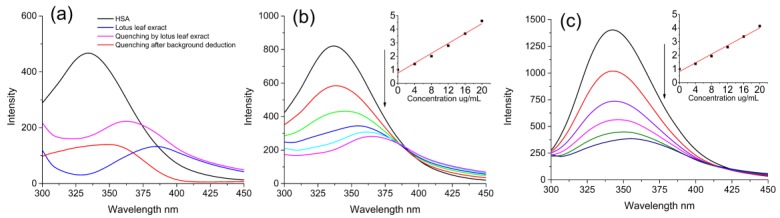
(**a**) The fluorescence spectra of human serum albumin (HSA) and lotus leaf extract; the quenching effects of lotus leaf extract on HSA fluorescence spectra and the Stern–Volmer plots for HSA (**b**) at 300.15 K; the quenching effects of lotus leaf extract on bovine serum albumin (BSA) fluorescence spectra and the Stern–Volmer plots for BSA (**c**) at 300.15 K. λ_ex_ = 280 nm; HSA and BSA, 10 μmol/L; the addition of sample was 4.00, 8.00, 12.00, 16.00 and 20.00 mg/L of lotus leaf extract.

The fluorescence spectra of HSA and BSA with addition of lotus leaf extracts were investigated and the results were shown in Figure 2. As shown in Figure 2a, the lotus leaf extract exhibited the emission of fluorescence at 390 nm (Blue line in Figure 2a) and it apparently quenched the fluorescence of HSA (Pink line in Figure 2a). According to the reduction of background fluorescence introduced by the extract, the fluorescence quenching of HSA and BSA with addition of lotus leaf extract in different concentrations were completed (Figure 2b,c). The Stern–Volmer plots for HSA and BSA fluorescence quenched by lotus leaf extract were also calculated (insets in Figure 2b,c). The Stern–Volmer plots were linear and the fitting degree (*R*^2^) was 0.976 for HSA and 0.983 for BSA. Considering the complex compositions of lotus leaf extract, the obtained fitting degrees were acceptable and detail investigations on 11 flavonoids were further conducted.

The fluorescence spectra of HSA and BSA with addition of apigenin, naringenin, luteolin, kaempferol, astragalin, quercetin, taxifolin, isorhamnetin, isoquercitrin, hyperoside, and rutin were measured. All tested samples could apparently quench the fluorescence of HSA and BSA with increasing concentrations. No obvious shifts of the maximum λ_em_ of HSA and BSA fluorescence for tested samples were observed. The decreases of fluorescence intensity varied with the kinds of samples. For instance, the addition of apigenin, kaempferol, and quercetin showed rapid quenching on HSA. The addition of apigenin made the fluorescence intensities of BSA decreased sharply. However, astragalin and rutin showed lower quenching rates on HSA and BSA. These results indicated that the quenching effect of flavonoids on HSA and BSA fluorescence depended on the structures of flavonoids [2].

**Figure 3:**
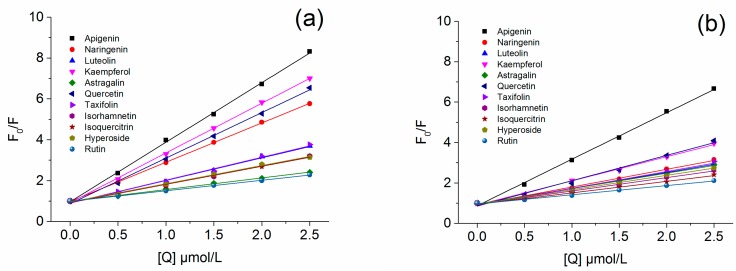
The Stern–Volmer plots for (**a**) HSA and (**b**) BSA fluorescences quenching by apigenin, naringenin, luteolin, kaempferol, astragalin, quercetin, taxifolin, isorhamnetin, isoquercitrin, hyperoside, and rutin at 300.15 K.

In order to further investigate the difference, the Stern–Volmer plots for HSA and BSA fluorescence quenched by flavonoids samples were studied and calculated. As shown in Figure 3, the Stern–Volmer plots of these flavonoids were linear and the fitting degree (*R*^2^) (ranging from 0.990 to 0.999) were satisfied, which implied that the calculations of *K*_sv_ and *K*_q_ according Equation (1) were suitable. The quenching could be considered initiated by static quenching if *K*_q_ is much greater than 2.0 × 10^10^ L/mol/s [42]. As shown in Table 2, the calculated *K*_q_ values of HSA and BSA fluorescence quenching by flavonoids were much higher than 2.0 × 10^10^ L/mol/s (0.827 × 10^14^ L/mol/s to 4.695 × 10^14^ L/mol/s for HSA and 0.731 × 10^14^ L/mol/s to 3.710 × 10^14^ L/mol/s for BSA). It could be concluded that the fluorescence quenching of HSA and BSA by flavonoids was probably initiated by static quenching [31].

### 2.3. The Binding Constants and the Number of Binding Sites

The binding constant (*K*_a_) and the number of binding sites per protein molecule (*n*) were calculated according to Equation (2) and shown in Table 2. The values of log *K*_a_ was proportional to the number of binding sites (*n*) for the interaction, which indicated that the Equation (2) used in this study was suitable for the investigation of interaction between flavonoids and serum albumins. The fitting degree (*R*^2^) (ranging from 0.993 to 0.999) of the calculated binding constant (*K*_a_) and the number of binding sites (*n*) were satisfied for this experiment. The magnitudes of apparent binding constants for HSA and BSA were respectively in the range of 10^5^ to 10^7^ L/mol and 10^6^ to 10^7^ L/mol.

### 2.4. Influence of Structural Alteration of Flavonoids on Their Affinities for HSA and BSA

#### 2.4.1. Hydroxylation and Methylation

The effects of hydroxylation and methylation of flavonoids on the affinities for HSA and BSA were shown in Table 3. It could be concluded that the hydroxylation on position 3 and/or 3’ of flavonoids improved the affinities for HSA and BSA. However, the methylation on the 3’ position of quercetin decreased the affinity as well. These results indicated that the hydroxyl group was particularly important for flavonoids in binding to HSA and BSA [3]. The result was in accordance with the previous report that the binding constants and the number of binding sites between flavonoids and HSA or BSA increased with the increased hydroxyl groups [43,44]. The affinities of luteolin and quercetin for HSA were about 7.9 and 6 times higher than that of apigenin and kaempferol.

#### 2.4.2. Glycosylation

The effect of glycosylation of flavonoids on the affinities for HSA and BSA were investigated. As shown in Table 3, the glycosylation at the 3 position of flavonoids decreased the affinities for HSA and BSA. The glycosylation of flavonoids decreased the affinities for serum albumins by 1 to 2 orders of magnitude, depending on the type of sugar moiety (glucoside, galactoside, and rutinoside). For instance, quercetin showed 3.20 times higher affinity than that of isoquercitrin, 1.94 times higher affinity than that of hyperoside and 15.60 times higher affinity than that of rutin in the interaction with HSA. While in the interaction with BSA, quercetin showed 1.60 times higher affinity than that of isoquercitrin, 2.56 times higher affinity than that of hyperoside, and 4.10 times higher affinity than that of rutin. This result was similar to the previous reports that the glycosylation of dietary polyphenols decreased the affinities for total plasma proteins [45].

#### 2.4.3. Hydrogenation of the C_2_=C_3_ Double Bond

The C_2_=C_3_ double bond in conjugation with a 4-oxo group plays an important role for the affinity for serum albumins [46]. As shown in Table 3, the hydrogenation of the C_2_=C_3_ double bond of flavonoids decreased the binding affinities for HSA and BSA. Both naringenin and taxifolin showed lower affinities than that of apigenin and quercetin. As mentioned by Xiao, the planarity of the C ring in flavonoids might be important for binding interaction with proteins. Because the molecules with saturated C_2_=C_3_ bonds permit more twisting of the B ring, resulting the harder enter of hydrophobic pockets in proteins [47,48].

### 2.5. Relationship of Topological Polar Surface Area (TPSA) and the Affinity for HSA and BSA

**Figure 4:**
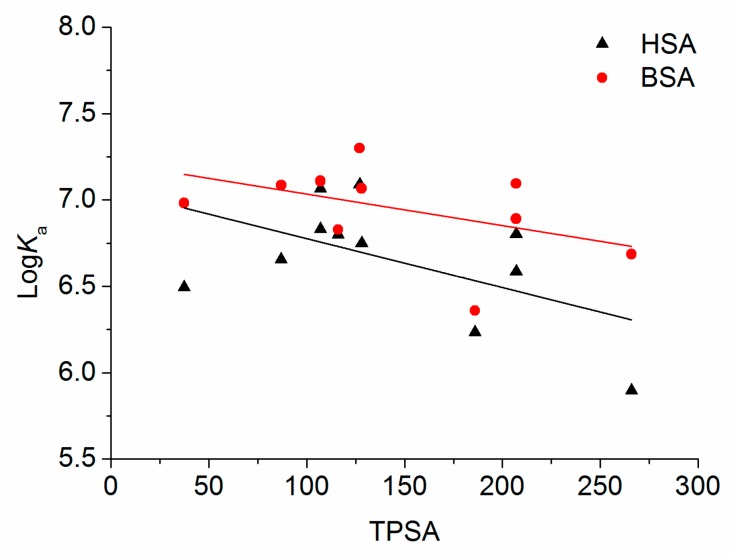
Relationship of TPSA with the affinities of flavonoids for HSA (black) and BSA (red).

As the sum of surfaces of polar atoms in a molecule, TPSA was a good indicator in the characterization of drug absorption, including intestinal absorption, bioavailability, and blood–brain barrier penetration. A strong correlation between TPSA and transport property could be found and the compound with high TPSA could be transported [44]. The TPSA values of tested flavonoids were confirmed from PubChem Public Chemical Database. The relationships between TPSA and binding affinity of flavonoids for HSA and BSA were plotted in Figure 4. As shown in Figure 4, no direct relationship between the TPSA values and log *K*_a_ values of flavonoids for HSA and BSA was found. However, it could be seen that the log *K*_a_ decreased with the increasing TPSA values of flavonoids for both HSA and BSA. The flavonoids with low TPSA were bound more tightly to HSA and BSA than those with high TPSA, which was in accordance with the reports [44,45].

## 3. Materials and Methods

### 3.1. Chemicals and Reagents

Human serum albumin (HSA) (96–99%, fraction V) and bovine serum albumin (BSA) (96%, fraction V) were purchased from GIBCO Co. (Shanghai, China). 3,5-dinitrosalicylic acid (DNS), potassium sodium tartrate tetrahydrate, soluble starch and 1,1-Diphenyl-2-picrylhydrazyl (DPPH) were acquired from Sigma-Aldrich Chemicals (St. Louis, MO, USA). Isorhamnetin (>98.0%), Apigenin (>98.0%), Naringenin (>98.0%), Kaempferol (>98.0%), Quercetin (>98.0%), Taxifolin (>98.0%), Luteolin (>96.0%), Isoquercitrin (>98.0%), Hyperoside (>98.0%), Astragalin (>98.0%), and Rutin (>98.0%) were purchased from Yuanye Biotechnology Co. (Shanghai, China). Ultrapure water (18.2 MΩ cm resistivity) was obtained from an ELGA water purification system (ELGA Berkefeld, Veolia, Germany). All of other chemicals were analytical grade and purchased from Sinopharm Chemical Reagent Co., Ltd. (Shanghai, China).

### 3.2. Preparation of Lotus Leaf Extracts

Dried lotus leaf (30.0 g) was powdered and extracted with 300 mL of 90% ethanol solution at 90 °C for 3 h to yield the crude extract [49,50]. Then, the extract solution was evaporated to remove the solvent under reduced pressure. The residue (3.18 g) was dissolved in 100 mL of methanol, filtered by a 0.45 μm membrane and stored at 4 °C prior to use.

### 3.3. Analysis of Flavonoids in Lotus Leaf Extracts

The analysis of 11 flavonoids in lotus leaf extract was conducted through HPLC analysis using an Agilent 1260 Infinity system (Agilent Technologies Inc., Santa Clara, CA, USA). A Waters Xbridge™ C_18_ reverse phase column (250 mm × 4.6 mm i.d., 5 μm) was used as the separation column (Waters, Milford, MA, USA). The column temperature was maintained at 25 °C and the flow rate was controlled at 0.8 mL/min. The mobile phase consisted of solvent A (water containing 0.1% *v*/*v* acetic acid) and solvent B (acetonitrile containing 0.1% *v*/*v* acetic acid) with gradient elution mode: 0–10 min, 15% B and 10–45 min, 15–45% B. The scan wavelength of diode array detector was set from 190–400 nm and the detection wavelength of chromatogram was set at 254 nm.

### 3.4. Protein Fluorescence Quenching Study

The fluorescence spectra were recorded on a Hitachi F-7000 fluorometer (Tokyo, Japan). The fluorescence quenching method was utilized to determine the binding constants between flavonoid and protein according to previous reports [6]. 20 μL of HSA or BSA solution (1.0 × 10^−5^ mol/L) in phosphate buffered saline (PBS, pH 7.4) was added into a 1.0 cm quartz cell and then flavonoids sample or lotus leaf extract (1.0 mmol/L or 4.0 mg/mL in methanol solution) was successively added manually with trace syringes. The intrinsic fluorescence emission spectra of HSA and BSA were recorded from 300 to 450 nm under an excitation wavelength of 280 nm. The fluorescence intensities were monitored at 336 nm for HSA and 341 nm for BSA and each test was performed in triplicate.

The fluorescence quenching of HSA and BSA by flavonoids was described by the Stern–Volmer equation shown as

F_0_/F = 1 + *K*_q_τ_0_[Q] = 1 + *K*_sv_[Q] (1)

where F_0_ is the fluorescence intensity of protein, F is the fluorescence intensity in the presence of flavonoids with different concentrations, [Q] is the corresponding concentration of flavonoids, *K*_q_ is the quenching rate constant, τ_0_ is the average lifetime (6.2 ns), and *K*_sv_ is the Stern–Volmer quenching constant.

For static quenching, after the fluorescence quenching intensities at 336 nm for HSA and 341 nm for BSA were measured, the relationship between fluorescence quenching intensity and the concentration of flavonoids could be described by the binding constant formula

log (F_0_ − F)/F = log *K*_a_ + *n*log[Q] (2)

where F_0_ is the fluorescence intensity of protein, F is the fluorescence intensity in the presence of flavonoids with different concentrations, [Q] is the corresponding concentration of flavonoids, *K*_a_ is the binding constant, and *n* is the number of binding sites per protein molecule. All the flavonoid samples showed no emission spectra in the scanned range under excitation.

## 4. Conclusions

The existence of 11 flavonoids in lotus leaf extract was confirmed in this study and 11 flavonoids showed various contents ranging from 0.063 ± 0.009 to 29.778 ± 0.180 mg/g in lotus leaf. The interactions between lotus leaf extract and serum albumins completed by spectroscopic methods showed apparent quenching effect on serum albumins. Then, the structure–affinity relationships of flavonoids on binding to serum albumins were further investigated. The hydroxylation on 3 and 3’ position improved the affinities for serum albumins. The methylation on 3’ position of quercetin, the C_2_=C_3_ double bonds of apigenin and quercetin and the glycosylations of quercetin and kaempferol all decreased the affinities for serum albumins. The results demonstrated that the hydrogen bond force played an important role in binding flavonoids to serum albumins.

## Figures and Tables

**Table 1:**
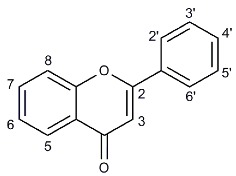
Chemical structures of investigated flavonoids and their contents in lotus leaf extract.

Flavonoids	Substitutions			Retention Time (min)	Content (mg/g)
	OH	OCH3	Others		
Apigenin	5,7,4′			38.84	0.216 ± 0.022
Naringenin	5,7,4′		Flavanone	39.46	1.446 ± 0.012
Luteolin	5,7,3′,4′			33.36	0.491 ± 0.039
Kaempferol	3,5,7,4′			40.27	0.063 ± 0.009
Astragalin	5,7,4′		3-*O*-glucoside	12.86	13.347 ± 0.150
Quercetin	3,5,7,3′,4′			33.77	2.628 ± 0.058
Taxifolin	3,5,7,3′,4′		Flavanone	20.41	10.597 ± 0.240
Isorhamnetin	3,5,7,4′	3′		40.92	0.390 ± 0.043
Isoquercitrin	5,7,3′,4′		3-*O*-glucoside	19.29	29.778 ± 0.180
Hyperoside	5,7,3′,4′		3-*O*-galactoside	18.58	3.292 ± 0.055
Rutin	5,7,3′,4′		3-*O*-rutinoside	17.25	1.588 ± 0.045

**Table 2:** The affinities of 11 flavonoids for human serum albumin (HSA) and bovine serum albumin (BSA).

Flavonoids	HSA Affinity						BSA Affinity					
	*K*_q_ (10^14^)	*K*_sv_ (10^6^)	*R*^2^	log*K*_a_	*n*	*R*^2^	*K*_q_ (10^14^)	*K*_sv_ (10^6^)	*R*^2^	log*K*_a_	*n*	*R*^2^
Apigenin	4.695	2.911	0.999	6.655	0.962	0.998	3.710	2.300	0.998	7.086	0.870	0.999
Naringenin	3.100	1.922	0.999	6.493	0.974	0.999	1.408	0.873	0.996	6.982	0.925	0.999
Luteolin	1.781	1.104	0.995	7.064	0.904	0.999	1.285	0.797	0.994	7.110	0.923	0.998
Kaempferol	3.923	2.432	0.999	6.830	0.918	0.999	1.923	1.192	0.996	7.106	0.911	0.995
Astragalin	0.918	0.569	0.997	6.234	0.994	0.993	1.224	0.759	0.997	6.359	0.963	0.998
Quercetin	3.594	2.228	0.998	7.089	0.868	0.999	2.018	1.251	0.991	7.299	0.858	0.999
Taxifolin	1.802	1.117	0.993	6.748	0.907	0.997	1.334	0.827	0.990	7.067	0.909	0.998
Isorhamnetin	1.445	0.896	0.994	6.798	0.923	0.999	1.066	0.661	0.995	6.828	0.941	0.999
Isoquercitrin	1.423	0.882	0.995	6.584	0.936	0.997	0.919	0.570	0.993	7.095	0.938	0.999
Hyperoside	1.434	0.889	0.996	6.802	0.960	0.995	1.148	0.712	0.997	6.891	0.950	0.996
Rutin	0.827	0.513	0.999	5.896	0.992	0.999	0.731	0.453	0.995	6.686	0.962	0.998

**Table 3:** Effects of hydroxylation, methylation, hydrogenation, and glycosylation of flavonoids on the affinities for HSA and BSA.

Structural Alteration	Examples	Effects (Times)	
		HSA Affinity	BSA Affinity
2,3-hydrogenation	Apigenin → Naringenin	1.45↓	1.27↓
	Quercetin → Taxifolin	2.19↓	1.71↓
3’H → OH	Apigenin → Luteolin	2.56↑	1.06↑
	Kaempferol → Quercetin	1.82↑	1.56↑
	Astragalin → Isoquercitrin	2.24↑	5.45↑
3H → OH	Apigenin → Kaempferol	1.50↑	1.05↑
3, 3’H → OH	Apigenin → Quercetin	2.72↑	1.63↑
	Naringenin → Taxifolin	7.80↑	1.22↑
3’OH → OCH_3_	Quercetin → Isorhamnetin	1.95↓	2.96↓
3-*O*-glycosylation	Quercetin → Isoquercitrin	3.20↓	1.60↓
	Quercetin → Hyperoside	1.94↓	2.56↓
	Quercetin → Rutin	15.60↓	4.10↓
	Kaempferol → Astragalin	3.94↓	5.58↓
